# Supplementary material for: A Post-Authorisation Safety Study of a Respiratory Syncytial Virus Vaccine in Pregnant Women and Their Offspring in a Real-World Setting: Generic Protocol for a Target Trial Emulation
Source: Vaccines (Basel). 2025 Mar 5;13(3):272. doi: 10.3390/vaccines13030272 (PMC11945849; doi:10.3390/vaccines13030272)
Supplement: Supplementary file 1 [file vaccines-13-00272-s001.zip › vaccines-3477821-supplementary.pdf]

SUPPLEMENTARY INFORMATION

A Post-Authorisation Safety Study of a Respiratory Syncytial Virus Vaccine in Pregnant Women and Their Offspring in a Real-World Setting: Generic Protocol for a Target Trial Emulation

Odette de Bruin [1,2,*], Linda Nab [1], Jungyeon Choi [1], Oisin Ryan [1], Hae-Won Uh [1], Fariba Ahmadizar [1], Shahar Shmuel [3], Heather Rubino [3], Kitty Bloemenkamp [2], Cynthia de Luise [3] and Miriam Sturkenboom [1]

[1] Department of Data Science & Biostatistics, Julius Global Health, University Medical Center Utrecht (UMCU), 3584CG Utrecht, The Netherlands; l.nab@umcutrecht.nl (L.N.); j.choi@umcutrecht.nl (J.C.); o.ryan@umcutrecht.nl (O.R.); haewonuh2013@gmail.com (H.-W.U.); f.ahmadizar@umcutrecht.nl (F.A.); m.c.j.sturkenboom@umcutrecht.nl (M.S.)

[2] Department of Obstetrics, Division Woman and Baby, Wilhelmina Children's Hospital, University Medical Center Utrecht (UMCU), 3584CG Utrecht, The Netherlands; k.w.m.bloemenkamp@umcutrecht.nl

[3] Safety Surveillance Research Worldwide Medical and Safety, Pfizer, Inc., New York, NY 10001-2192, USA; shahar.shmuel@pfizer.com (S.S.); heather.rubino@pfizer.com (H.R.); cynthia.deluise@pfizer.com (C.d.L.)

* Correspondence: o.debruin-3@umcutrecht.nl

Supplementary Table 1. Risk factors for the outcomes of interest

PRETERM BIRTH[1,2]	
**Risk factors**	**Covariate assessment window**
Advanced maternal age (≥35 years) or early maternal age (<20 years)	At time of vaccination
Race/ethnicity: African American, Afro-Caribbean	From database entry to time of vaccination
Smoking during pregnancy, substance abuse/dependence during pregnancy	From start of pregnancy to time of vaccination
This pregnancy: nulliparity, multifetal pregnancy, ART, short uterine cervix (<25 mm in second trimester), gestational hypertension, preeclampsia, gestational diabetes, premature rupture of membrane, placenta previa, antepartum haemorrhage	From start of pregnancy to time of vaccination
Prior pregnancy history of: preterm birth, abortion, caesarean delivery	From database entry to time of vaccination
Birth space less than 2 years	From database entry to time of vaccination
Morbidities: anaemia, asthma, obesity (pre-pregnancy BMI >30 kg/m2), pre-existing diabetes, chronic hypertension, depression, thyroid disease, anomalies of the uterus (e.g., presence of a uterine septum)	Chronic comorbidities: From database entry to time of vaccination. Time-varying comorbidities: From 365 days prior to time of vaccination.
Infections: HIV, urinary tract infections, chlamydia, toxoplasmosis, trichomonas vaginalis, malaria, COVID-19	Chronic infections: From database entry to time of vaccination. Acute infections: From start pregnancy to time of vaccination.
Medicines: antidepressants, benzodiazepines, selective serotonin receptor inhibitors, antibiotics, NSAIDs	From start of pregnancy to time of vaccination
**STILLBIRTH[3,4]**	
**Risk factors**	**Covariate assessment window**
Advanced maternal age (≥35 years) or early maternal age (<20 years)	At time of vaccination
Race/ethnicity: Black, African American	From database entry to time of vaccination
Smoking, substance abuse/dependence	From start of pregnancy to time of vaccination
This pregnancy: nulliparity, multifetal pregnancy, ART, gestational hypertension, preeclampsia, gestational diabetes, FGR, congenital anomaly, premature rupture of membrane, antepartum haemorrhage, placental abruption	From start of pregnancy to time of vaccination
Prior pregnancy history of: stillbirth, pregnancy loss, miscarriage, preterm birth, SGA	From database entry to time of vaccination
Morbidities: obesity (pre-pregnancy BMI >30 kg/m^2), pre-existing diabetes, chronic hypertension, chronic kidney disease, thyroid disorders, SLE, sickle cell disease	Chronic comorbidities: From database entry to time of vaccination. Time-varying comorbidities: From 365 days prior to time of vaccination.
Infections: HIV, Escherichia coli, Klebsiella, Group B Streptococcus, Enterococcus, Mycoplasma/Ureaplasma, Haemophilus influenzae, Chlamydia	Chronic infections: From database entry to time of vaccination. Acute infections: From start pregnancy to time of vaccination.
**HYPERTENSIVE DISORDERS OF PREGNANCY[5,6]**	
**Risk factors**	**Covariate assessment window**
Advanced maternal age (≥40 years)	At time of vaccination
Race/ethnicity: African American	From database entry to time of vaccination
Smoking (protective)	From start of pregnancy to time of vaccination
This pregnancy: nulliparity, multifetal pregnancy, ART	From start of pregnancy to time of vaccination
Prior pregnancy history of: pre-eclampsia, placental abruption, stillbirth, FGR	From database entry to time of vaccination
Morbidities: obesity (pre-pregnancy BMI >30 kg/m^2), pre-existing diabetes, chronic hypertension, chronic kidney disease (inc. kidney transplanted women), SLE antiphospholipid antibody syndrome, rheumatoid arthritis, sickle cell disease, PCOS, multiple sclerosis	Chronic comorbidities: From database entry to time of vaccination. Time-varying comorbidities: From 365 days prior to time of vaccination.
Infections: urinary tract infections	Chronic infections: From database entry to time of vaccination. Acute infections: From start pregnancy to time of vaccination.

[1] NIH. What are the risk factors for preterm labor and birth? 2023. Available at: https://www.nichd.nih.gov/health/topics/preterm/conditioninfo/who_risk. Accessed 8 April 2024.
[2] Quinn J-A, Munoz FM, Gonik B, et al. Preterm birth: Case definition & guidelines for data collection, analysis, and presentation of immunisation safety data. Vaccine **2016**; 34:6047–6056.
[3] Da Silva FT, Gonik B, McMillan M, et al. Stillbirth: Case definition and guidelines for data collection, analysis, and presentation of maternal immunization safety data. Vaccine 2016; 34:6057. Available at: /pmc/articles/PMC5139804/. Accessed 5 April 2024.
[4] NIH. What are the risk factors for stillbirth? 2023. Available at: https://www.nichd.nih.gov/health/topics/stillbirth/topicinfo/risk. Accessed 8 April 2024
[5] Magee LA, Brown MA, Hall DR, et al. The 2021 International Society for the Study of Hypertension in Pregnancy classification, diagnosis & management recommendations for international practice. Pregnancy Hypertens 2022; 27:148–169. Available at: https://pubmed.ncbi.nlm.nih.gov/35066406/. Accessed 5 April 2024.
[6] NIH. Who is at risk of preeclampsia? Available at: https://www.nichd.nih.gov/health/topics/preeclampsia/conditioninfo/risk. Accessed 8 April 2024.

Supplementary Table 1 (continued). Risk factors for the outcomes of interest

**GUILLIAN-BARRÉ SYNDROME**[7,8]	
**Risk factors**	**Covariate assessment window**
Co-morbidities: HIV, mycoplasma pneumonia	Chronic comorbidities: From database entry to time of vaccination. Time-varying comorbidities: From 365 days prior to time of vaccination.
Infections: Campylobacter jejuni, CMV, EBV, influenza, Zika virus	Chronic infections: From database entry to time of vaccination. Acute infections: From start pregnancy to time of vaccination.
**LOW BIRTH WEIGHT**[9,10]	
**Risk factors**	**Covariate assessment window**
Advanced maternal age (≥35 years) or early maternal age (<20 years)	At time of vaccination
Race/ethnicity: Black	From database entry to time of vaccination
Smoking, substance abuse/dependence	From start of pregnancy to time of vaccination
This pregnancy: nulliparity, multifetal pregnancy, preterm birth, gestational hypertension, preeclampsia, gestational diabetes, FGR, congenital anomaly	From start of pregnancy to time of vaccination
Prior pregnancy history of: preterm birth, low birth weight	From database entry to time of vaccination
Morbidities: anaemia, pre-existing diabetes, chronic hypertension, chronic kidney disease	Chronic comorbidities: From database entry to time of vaccination. Time-varying comorbidities: From 365 days prior to time of vaccination.
Infections: HIV, CMV, rubella, chickenpox, toxoplasmosis, chlamydia	Chronic infections: From database entry to time of vaccination. Acute infections: From start pregnancy to time of vaccination.
**SMALL FOR GESTATIONAL AGE**[11,12,13]	
**Risk factors**	**Covariate assessment window**
Advanced maternal age (≥35 years) or early maternal age (<20 years)	At time of vaccination
Race/ethnicity: Black	From database entry to time of vaccination
Smoking, substance abuse/dependence	From start of pregnancy to time of vaccination
This pregnancy: nulliparity, multifetal pregnancy, ART, gestational hypertension, preeclampsia, gestational diabetes, FGR, congenital anomaly, placental abruption	From start of pregnancy to time of vaccination
Prior pregnancy history of: SGA	From database entry to time of vaccination
Morbidities: anaemia, pre-existing diabetes, chronic hypertension, chronic kidney disease, SLE, sickle cell disease, anomalies of the uterus (e.g., presence of a uterine septum)	Chronic comorbidities: From database entry to time of vaccination. Time-varying comorbidities: From 365 days prior to time of vaccination.
Infections: HIV, CMV, rubella, toxoplasmosis	Chronic infections: From database entry to time of vaccination. Acute infections: From start pregnancy to time of vaccination.

Abbreviations: ART, assisted reproductive technology; BMI, body mass index; CMV, cytomegalovirus; EBV, Epstein Barr virus; FGR, foetal growth restriction; GBS, Guillain-Barré syndrome; HIV, human immunodeficiency viruses; NSAIDS, non-steroidal anti-inflammatory drugs; PCOS, polycystic ovary syndrome; SGA, small for gestational age; SLE, systemic lupus erythematosus.

[7] WHO. Guillain–Barré syndrome. 2023. Available at: https://www.who.int/news-room/fact-sheets/detail/guillain-barr%C3%A9-syndrome. Accessed 8 April 2024.
[8] Sejvar JJ, Kohl KS, Gidudu J, et al. Guillain-Barré syndrome and Fisher syndrome: case definitions and guidelines for collection, analysis, and presentation of immunization safety data. Vaccine 2011; 29:599.
[9] De Bernabé JV, Soriano T, Albaladejo R, et al. Risk factors for low birth weight: a review. European Journal of Obstetrics & Gynecology and Reproductive Biology 2004; 116:3–15.
[10] Cutland CL, Lackritz EM, Mallett-Moore T, et al. Low birth weight: Case definition & guidelines for data collection, analysis, and presentation of maternal immunization safety data. Vaccine 2017; 35:6492.
[11] De Bernabé JV, Soriano T, Albaladejo R, et al. Risk factors for low birth weight: a review. European Journal of Obstetrics & Gynecology and Reproductive Biology 2004; 116:3–15.
[12] MSD manual. Small-for-Gestational-Age (SGA) Newborns. 2024. Available at: https://www.msdmanuals.com/home/children-s-health-issues/general-problems-in-newborns/small-for-gestational-age-sga-newborn. Accessed 8 April 2024.
[13] Schlaudecker EP, Munoz FM, Bardají A, et al. Small for gestational age: Case definition & guidelines for data collection, analysis, and presentation of maternal immunisation safety data. Vaccine 2017; 35:6518. Available at: /pmc/articles/PMC5710996/. Accessed 5 April 2024.

Supplementary Table 2. Power and sample size estimates for Guillain-Barré Syndrome and pregnancy and birth outcomes

**Risk Ratio (RR)**	**RR=1.5**	**RR=2.0**	**RR=2.5**	**RR=4.0**
**Guillain-Barré Syndrome**				
Incidence rate 1/100,000 person/years, maximum number exposed 30,000, 1:1 ratio unexposed:exposed				
Power to detect	0.0341	0.0406	0.0461	0.0628
**Pregnancy and birth outcomes**				
Power of 80%, 1:1 ratio unexposed:exposed, rounded-up to nearest hundred				
Prevalence 0.2% (e.g., stillbirth)	38600	11300	5700	1900
Prevalence 1%	7600	2300	1200	400
Prevalence 2%	3800	1100	600	200
Prevalence 3%	2500	800	400	100
Prevalence 4%	1900	600	300	100
Prevalence 5%	1500	500	300	100
Prevalence 6% (e.g., preterm birth, low birth weight)	1200	400	200	100

Based on the Cohort Power tab of episheet by K. Rothman and O. Miettinen[14]

[14] KJ Rothman. Episheet . Available at: http://krothman.hostbyet2.com/Episheet.xls. Accessed 14 May 2024.
